# Shared humanity, awareness of socio-economic privilege, and classism during the pandemic as predictors of supporting equal socio-economic policies

**DOI:** 10.1007/s12144-021-01734-3

**Published:** 2021-04-30

**Authors:** Özden Melis Uluğ, Nevin Solak, Betül Kanık

**Affiliations:** 1 Department of Psychology, Clark University, 950 Main Street, Worcester, MA 01610 USA; 2 University of Sussex, Brighton, UK; 3 TED University, Ankara, Turkey; 4 Hacettepe University, Ankara, Turkey

**Keywords:** Shared humanity, Class-based privilege, Classism, Coronavirus, Socio-economic policies, Pandemic

## Abstract

The coronavirus pandemic has caused unemployment to skyrocket, exposed the longstanding inequalities in health care services and working conditions, and mainly affected the poor in different parts of the world. In the current study, we focus on social identity and social class-related factors that are critical during the pandemic to gain insights into what predicts support for policies favoring economic equality in the post-pandemic period. We argue that to the extent that individuals 1) identify with all humanity during the pandemic, 2) are aware of their socio-economic status-based privilege, 3) do not hold classist attitudes, they would support policies favoring economic equality. In Study 1, survey data from 1212 participants in Turkey were analyzed by means of hierarchical linear regression analysis. The findings showed that stronger identification with all humanity, higher awareness of socio-economic status-based privilege, and less endorsement of classist attitudes predict more support for socio-economic equality policies in the post-pandemic period, after controlling for socio-demographic and socio-political characteristics of participants. Study 2 (*N* = 212) replicated the findings in a different context, namely the U.S. Our findings extend previous studies by showing the importance of a global identity, such as shared human identity, in the ongoing and potentially in the aftermath of the pandemic. In addition, our findings highlight the joint contributions of socio-economic factors such as classist attitudes and awareness of class-based privilege to the support for socio-economic policies.

The economic gap between the poorest and the wealthiest tends to increase in the aftermath of most disasters (Jetten et al., 2017). The coronavirus pandemic has caused unemployment to skyrocket and exposed the longstanding inequalities in health care services and working conditions, which have mainly affected the poor in different parts of the world (e.g., Jetten et al., 2020; Reeves & Rothwell, 2020). In the current study, we draw on social identity and social class-related factors that are critical during the pandemic in an attempt to gain insights into what predicts support for policies favoring economic equality in the post-pandemic period. Identifying the factors contributing to supporting economic equality is crucial for understanding facilitators and barriers to promoting social justice. Specifically, in order to understand the dynamics behind support for equal socio-economic policies, social psychologists need to identify factors that may play roles in supporting equal socio-economic policies.

One such factor that can advance economic equality is shared humanity. Research on superordinate categorization has demonstrated that increasing category inclusiveness (from the group level to the human level) yielded some positive consequences for improving attitudes between groups, even in contexts of conflict (e.g., Greenaway, Quinn, & Louis, 2011; Wohl & Branscombe, 2005; but see Uluğ et al., 2021). The coronavirus pandemic has inspired discussions about how it unites peoples and communities worldwide (Tekin et al., 2021) through shared human identity. Shared humanity can hold large groups together and shape group members’ willingness to act in ways that increase the likelihood of positive collective outcomes (see Jetten et al., 2020). We, therefore, argue that to the extent that individuals identify with all humanity during the pandemic, they are more likely to support policies favoring economic equality in the post-pandemic period.

As the pandemic has progressed, it also has led to acts of division and discrimination (Malik, 2020). Some have blamed poor people for increasing the virus’s transmission and being a burden on their health and economic systems (Diallo, 2020; Waugh, 2020). Each of these examples speaks to the fact that the coronavirus pandemic has enhanced classism such that negative attitudes, beliefs, and behaviors are directed toward those from low social class (Lott, 2012). In addition to classism, awareness of class-based privilege also plays an important role in supporting certain economic policies. Research on privilege awareness in the context of intergroup relations has indicated that among advantaged groups, awareness of privilege may be an important motivating factor in supporting disadvantaged groups, both in favoring policies that improve conditions for the disadvantaged group, and in participating in collective action on behalf of the disadvantaged group (e.g., Stewart et al., 2012; Uluğ & Tropp, 2021). We argue that in the aftermath of the pandemic, individuals with classist beliefs would be less likely to support economic policies promoting socio-economic equality, while individuals with a heightened class-based awareness would have a greater likelihood of supporting such policies.

In the current research, we focus on the role of shared humanity, especially during emergencies such as a pandemic, in predicting support for equal socio-economic policies. We also focus on socio-economic factors such as how awareness of class-based privilege, as well as classist attitudes during the coronavirus pandemic, may play an important role in people’s support for equal socio-economic policies. We test these predictions in two different societal contexts: Turkey and the U.S. We aim to extend previous studies by (a) showing the importance of a global identity such as shared human identity in the ongoing and aftermath of a pandemic, and (b) examining the joint contributions of socio-economic beliefs such as people’s awareness of class-based privilege and classism to supporting equal socio-economic policies.

## Shared Humanity during the Coronavirus Pandemic

Shared humanity can be defined as a global identity that encompasses all human beings and that sees all humanity as a family (McFarland, 2010). It also encompasses “a deep caring for all human beings regardless of their race, religion, or nationality” and it is more than “the sum of positive qualities, such as dispositional empathy and principled moral reasoning” (McFarland, Brown, & Webb, 2013, p. 194). In the literature, this term is used interchangeably with *one humanity* (e.g., McFarland et al., 2013), *identification with all humanity* (e.g., Hamer, McFarland, & Penczek, 2019; McFarland, Webb, & Brown, 2012; Reese, Proch, & Finn, 2015), *global identity/citizen* (e.g., Barth et al., 2015; Reese & Kohlmann, 2015; Reysen & Katzarska-Miller, 2013), *world community* (e.g., Reese, Proch, & Cohrs, 2014), *the world as a whole* (e.g., Buchan et al., 2011), *citizen of the world* (e.g., Türken & Rudmin, 2013), *cosmopolitan identity* (e.g., Türken & Rudmin, 2013) and *global belonging* (e.g., Der-Karabetian, Cao, & Alfaro, 2014).

The shared humanity concept has received considerable attention in social psychology in recent years. It has become a popular topic as it focuses both on harmonious intergroup relations and caring for all humanity (Reese & Kohlmann, 2015). It has been studied in many populations and contexts, including Germany (Reese et al., 2014; Reese & Kohlmann, 2015; Renger & Reese, 2017; Römpke, Fritsche, & Reese, 2019), the U.S. (Chayinska et al., 2021; Hamer et al., 2021; McFarland et al., 2012; Reysen & Hackett, 2016; Reysen & Katzarska-Miller, 2013; Türken & Rudmin, 2013), Norway (Türken & Rudmin, 2013), Canada (Lee et al., 2015), Turkey (Türken & Rudmin, 2013), Poland (Hamer et al., 2019; Hamer et al., 2021), France, Mexico, Chile (Hamer et al., 2021), Italy, Russia, Argentina, South Africa, and Iran (Buchan et al., 2011; Chayinska et al., 2021).

Previous studies have focused on examining the antecedents of identification with all humanity (IWAH) for individuals, intergroup relations, and societies on the one hand and outcomes of identifying with all humanity on the other. To give a few examples, higher openness to experience, empathy, and universalism-tolerance have been found as antecedents of increased identification with all humanity (Hamer et al., 2019; see also Lee et al., 2015). In addition, it has been shown that participation in both formal and informal experiential learning opportunities, such as study abroad opportunities, increases people’s identification with global identities (Scott Belt, 2016). On the other hand, IWAH is also positively associated with concern for global issues and prosocial attitudes (McFarland et al., 2012), such as support for refugees and asylum seekers (Bassett & Cleveland, 2019; Nickerson & Louis, 2008), support for ending global hunger (McFarland et al., 2019), support for human rights and humanitarian reliefs (McFarland et al., 2012), support for Fairtrade products over conventional products (Reese & Kohlmann, 2015), and support for environmental sustainability (Reysen & Katzarska-Miller, 2013).

The coronavirus pandemic has affected all humanity, and it can be seen as a shared challenge experienced by all humankind. This shared challenge may help bring all people together to cope with the problems we are facing effectively due to the pandemic. The solution to crises like the coronavirus pandemic requires global cooperation. As such, people’s willingness to support each other, especially the disadvantaged other, depends on their inclusion of the disadvantaged others in them*selves* (Römpke et al., 2019). In this context, identification with all humanity may provide researchers a framework to explain how responses to this global challenge may differ (see, e.g., Hamer et al., 2021, for a discussion). It is fair to argue that identification with all humanity may help people endorse the beliefs that social and economic inequalities in today’s society are unjust, and everyone is fundamentally deserving of similar positive outcomes, which thus may inform political reactions amidst global challenges (see, e.g., Reese & Kohlmann, 2015). As the coronavirus pandemic exposed the longstanding inequalities in societies, mainly affected the individuals from a low socio-economic background in different parts of the world (e.g., see Jetten et al., 2020; Reeves & Rothwell, 2020), we examine whether identification with all humanity during the coronavirus pandemic predict people’s support for equal socio-economic policies. Given that people’s awareness of interconnectedness with others may play an important role in endorsing prosocial values through a greater connection with global citizens (cf. Reysen & Katzarska-Miller, 2013) and such global identity is more likely to generate solidarity with disadvantaged groups (Barth et al., 2015; but see Chayinska et al., 2021), we expect that the more people identify with all humanity, the more they support these equal socio-economic policies favoring economic equality in the post-pandemic period.

## Classism and Support for Socio-Economic Policies

Classism can be defined as prejudice and discrimination based on social class (see Kite & Whitley, 2016). Lott (2002) argues that distancing is the dominant response to the poor and that distancing, separation, exclusion, and devaluation, together with stereotyping and prejudice, define classism. Classism can occur when negative beliefs and stereotypes about low-income groups are cited frequently (Langhout, Rosselli, & Feinstein, 2007). Numerous research has demonstrated that poor individuals are often viewed as lazy and irresponsible by individuals who are not poor (Cozzarelli, Tagler, & Wilkinson, 2002; Feagin, 1975; Furnham, 1982; Lott, 2002). Moreover, it has been shown that some of the poor may internalize classism by making victim-blaming attributions for poverty, such as lack of responsibility and personal failure (Hunt, 1996; Kluegel & Smith, 1986; Napier et al., 2006).

Classism has some consequences for supporting socio-economic policies. For example, individualistic attributions for poverty are associated with support for reduced funding, restrictive, and conservative policies (Appelbaum, 2001; Bullock, 1999; Bullock, 2008; Bullock, Williams, & Limbert, 2003; Kluegel & Smith, 1986). As the pandemic has progressed, it has led to increased prejudice relative to social class (e.g., Rosen, 2020). For example, some have blamed low-income groups for increasing the virus’s transmission and have seen them as a burden on their health and economic systems (Diallo, 2020; Malik, 2020; Waugh, 2020). Each of these examples shows enhanced classism during the coronavirus pandemic. Yet, classism’s role in reactions toward economic policies has remained understudied. We argue that classist attitudes in the coronavirus pandemic context would negatively predict support for equal socio-economic policies in the post-pandemic period.

## Economic Privilege Awareness and Support for Socio-Economic Policies

Another important factor relevant to economic policy preferences is the enhancement of dominant group members’ awareness of the illegitimate advantages they have in society, or in our study context, socio-economic status-based privilege. Privilege is typically defined as unearned status and benefits in reference to social identities, leading to advantages, opportunities, benefits, and access to resources among those who have these identities (e.g., Black & Stone, 2005; Taiwo, 2018). Privilege awareness is the awareness of having unearned status and benefits because of one’s social group. Research on privilege awareness has mostly focused on race and gender (see, e.g., Case, 2012), while socio-economic privilege is an understudied concept (but see Côté et al., 2021; Dunlap et al., 2007). Socio-economic status (SES) refers to differential access (potential and realized) to desired resources (Oakes & Rossi, 2003). Much like race- and gender-based privilege, a privileged status based on one’s SES provides status, power, and rank to those who occupy this position, and it leads to greater access to educational, health care, economic and social benefits in society (Black & Stone, 2005).

Research on privilege awareness in different contexts of intergroup relations indicated that privilege awareness among advantaged groups might motivate them to support policies to improve disadvantaged groups’ conditions. For example, heightened racial privilege awareness among White Americans has been shown to predict the presence of more positive attitudes toward African Americans (Stewart et al., 2012) and reduced attitudes of racism (Powell, Branscombe & Schmitt, 2005). Relatedly, among heterosexuals, greater acknowledgment of heterosexual privilege was associated with the development of more positive attitudes toward same-sex marriage and marriage equality (Case & Stewart, 2010).

In the context of COVID-19, the privileged are less vulnerable than the poor to suffering brought about by the virus (see Jetten et al., 2020). For example, it is evident that the economically privileged are more likely to be able to stay home, have secure employment, and better access to health care than the poor. Consequently, such benefits and opportunities held by economically privileged groups may become more visible in the pandemic. Despite the increased social-economic status-based discrepancies in societies during the pandemic, we know little about the ways in which economic privilege awareness plays a role in reactions toward socio-economic policies. Following the literature on privilege awareness, we expect that heightened socio-economic status-based privilege awareness in the coronavirus pandemic context would positively predict support for equal socio-economic policies in the post-pandemic period.

## Overview of Studies

In the current studies, we aimed to investigate the role of identification with all humanity and socio-economic beliefs, which emerged as critical factors during the pandemic, in supporting policies related to socio-economic equality. Following the literature on the relevant concepts, we formulated three hypotheses. We hypothesized that people would support equal socio-economic policies favoring economic equality in the post-pandemic period to the extent they identify with all humanity in a context of a global crisis that affects all people (*Hypothesis 1*). In addition, we hypothesized that people’s classist attitudes would negatively predict their support for economic equality (*Hypothesis 2*). In contrast, their awareness of class-based privilege would positively predict their support for economic equality (*Hypothesis 3*). Given investigating these hypotheses in cross-country contexts may help researchers find global solutions in tackling socio-economic problems, we tested these relationships in two different contexts: Turkey (Study 1) and the United States (Study 2).

## Study 1

### Method

#### Participants and Procedure

We received IRB approval for this research from Clark University. Participants were recruited via snowball convenience sampling over social media (i.e., Facebook and Twitter) in April 2020 and completed the survey voluntarily. At the beginning of the study, participants were informed about the purpose of the study, were told that they could withdraw from the study at any time during data collection without having to give a reason, and there is no potential risk to participants. Participants gave their informed consent prior to their inclusion in the study.

The original sample consisted of 1309 participants from Turkey. Participants were rejected for: failing attention check question^1^ (*N* = 78) and being younger than 18 (*N* = 19). After implementing these filters, the remaining sample consisted of 1212 participants. Out of 1212 participants, 906 self-identified as Turk, 91 as Kurd, 29 as Circassian, 13 as Arab, 163 as other, and 10 did not respond to this question.

Seven hundred five participants self-identified as women, 500 as men, six as “other,” and one did not respond. Participants’ ages ranged from 18 to 74 (*M* = 31.53; *SD* = 9.40). Two hundred ninety-three participants completed an MSc/PhD degree, 582 a university degree (4 years), 62 a university degree (2 years), 267 high school, seven secondary school, and one primary school. When describing their income, 16 (1%) participants chose *less than 1000 TL*, 159 (13%) *1001–3000 TL*, 246 (21%) *3001–5000 TL*, 242 (20%) *5001–7500 TL*, 223 (19%) *7501–10,000 TL*, 162 (14%) *10,001–15,000 TL*, 96 (8%) *15,001–25,000 TL*, 55 (5%) *more than 25,001 TL* and 13 participants did not respond to this question.

When describing their socio-economic status, on a 7-point scale (1 = *low SES*; 7 = *high SES*), 33 (3%) participants chose 1, 100 (8%) 2, 318 (26%) 3, 449 (37%) 4, 257 (21%) 5, 43(4%) 6, and 12 (1%) 7. When asked to describe their political orientation on a scale from 1 (*left*) to 9 (*right*), respondents indicated a wide variety of responses across the political spectrum (*M* = 3.09; *SD* = 1.61).

#### Measures

The scales were presented in random order. With the exception of the demographic variables mentioned above, all items used 7-point response scales (1 = *strongly disagree/not at all*, 7 = *strongly agree/very much*). Descriptive statistics are reported in Table 1. Since our measures were newly constructed or adjusted, to evaluate the viability of the one-factor structure of each scale, we conducted confirmatory factor analyses using IBM Statistics AMOS 23 (Arbuckle, 2014) on data from 1212 participants. According to conventional criteria (see Kline, 2005), a good fit is reflected by the root mean square error of approximation (*RMSEA*) is lower than 0.08, and the Comparative Fit Index (*CFI*), Goodness of Fit Index (*GFI*), Adjusted Goodness of Fit Index (*AGFI*), Bentler-Bonett Non-Normed Fit Index (*NNFI*) are around 0.90. We employed variance-covariance matrices as inputs and used maximum-likelihood method.

**Table 1:** Means, Standard Deviations, and Correlations among the Study Variables in Study 1

Variables	*M (SD)*	1	2	3	4	5	6	7	8	9	10
1. Gender (1 = *Female,* 2 = *Male*)	–	–	.040	.052	−.100**	−.035	.088**	−.117***	.028	.113***	−.084**
2. Age	31.53 (9.39)		–	.314***	.424***	.187***	−.129***	.163***	.027	−.029	.079**
3. Income	4.37 (1.70)			–	.292***	.585***	.018	.109***	−.044	.083**	−.027
4. Education	6.73 (1.08)				–	.226***	−.092**	.095**	.074**	−.026	.040
5. Subjective SES	3.80 (1.11)					–	.060*	.068*	−.161***	.102***	−.047
6. Political Ideology	3.09 (1.61)						–	−.033	−.330***	.154***	−.233***
7. Shared Humanity	4.56 (1.43)							–	−.104***	−.092**	.150***
8. Awareness of Socio-economic Privilege	5.82 (1.08)								–	−.091**	.195***
9. Classism	1.67 (0.86)									–	−.409***
10. Support for Policies about Socio-economic Equality	6.33 (0.69)										–

*Note*. ***p < .001, **p < .01, *p < .05

##### Identification with all Humanity

We measured shared humanity via identification with all humanity scale in the context of the coronavirus pandemic. We used four items inspired by Buchan et al. (2011) and Reysen and Katzarska-Miller (2013): *Considering this pandemic period*, “*I think I share a similar fate with everyone in the world these days*,” “*These days I feel closer to everyone in the world*,” “*These days I have realized that there are many similarities between myself and people around the world*,” and “*I see myself as a world citizen these days*” (Cronbach’s *α* = 0.81). We tested a model in which identification with humanity items loaded on a single factor. The fit indices for the model were as follow: χ2(2, *N* = 1212) = 20.734, *p* < .001, *RMSEA* = .088, *GFI* = .991*, AGFI* = .957, *CFI* = .988. Item factor loadings were between .64 and .79.

##### Awareness of Socio-Economic Privilege

We created five items to measure awareness of socio-economic privilege during the coronavirus pandemic period: *In this pandemic period, “in our society, people are valued as much as their socio-economic status*,” “*those with good financial conditions are treated as privileged in our health care system*,” “*in general, those with good economic conditions in our society have many advantages (e.g., access to food, using health services, access to school education, etc.) and privileges that the poor do not have*,” “*in our society, the health of those who are in good financial condition is seen as more important than the health of those who are not financially good*,” and “*our health care system works in a way that individuals are valued as much as their socio-economic status*” (Cronbach’s *α* = 0.85). We examined whether all the items in the measure loaded on the same factor. The model fit indices indicated an acceptable fit, except the *RMSEA* value, χ2(5, *N* = 1212) = 96.033, *p* < .001, *RMSEA* = .123, *GFI* = .967, *AGFI* = .901, *CFI* = .966. Item factor loadings were between .47 and .85.

##### Classism

We created three items in the context of the pandemic inspired by North and Fiske’s (2013) work on ageism: *In this pandemic period*, “*those with low socio-economic status are a huge burden on our health system*,” “*those with low socio-economic status are a burden rather than contributing to society*,” and “*those with poor socio-economic status should receive the same respect in society as they are with a good socio-economic status*” (reverse coded; Cronbach’s *α* = 0.68). We assessed whether all the items in the measure loaded on the same factor, goodness of fit statistics were as follow: χ2(0, *N* = 1212) = 0.00, *CFI* = 1.000. Item factor loadings were between −.35 and .84.^2^

##### Support for Policies about Socio-Economic Equality

We created five items to assess support for policies about socio-economic equality: *Over the next few years, I support* “*social policies for rich and poor students to study in the same classrooms and schools*,” “*social policies aimed at reducing economic status differences in health care*,” “*social policies for the rich and the poor to benefit from the same health services*,” “*policies to reduce the privileged position of those who are in good financial situations (e.g., education, health, work-life, etc.)*” and “*the state’s budget allocation to improve the living conditions of those who are not financially good*” (Cronbach’s *α* = 0.70). We, again, run confirmatory factor analysis. The single factor model shows an acceptable fit, *χ*2(5, *N* = 1212) = 25.563, *p* < .001, *RMSEA* = .058, *GFI* = .992, *AGFI* = .975, *CFI* = .986. Item factor loadings were between .43 and .77.

##### Socio-Demographic and Socio-Political Questions

We asked participants’ gender, age, income, education, subjective socio-economic status (SES), and political ideology.

### Results and Discussion

Table 1 presents correlations and other descriptive statistics in Study 1. As only gender, age, and political ideology significantly correlated with support for policies about economic equality, they were included in regression analysis. A hierarchical regression analysis^3^ was carried out in SPSS version 26 (IBM Corp, 2017) to examine the degree to which (a) socio-demographic and socio-political characteristics of participants (gender, age, and political ideology), (b) shared humanity (identification with all humanity), and c) beliefs about socio-economic inequality (classism and awareness of socio-economic privilege) predicted support for policies about socio-economic equality. In this analysis, three socio-demographic and socio-political variables were entered in Step 1 as control variables, followed by the shared humanity variable in Step 2. The two variables related to beliefs about socio-economic inequality were added in Step 3. The standardized and unstandardized coefficients of our analyses are presented in Table 2.

**Table 2:** Model of Summary of Regression Analysis in Study 1

Support for Policies about Socio-economic Equality					
	Unstandardized β	Standardized *β*	*t*	*R*^2^	Δ*R*^2^
**Step 1**				.058	.058
Gender (1 = *Female*, 2 = *Male*)	−.091*	−.066*	−2.347		
Age	.004	.053	1.876		
Political Ideology	−.092***	−.214***	−7.554		
**Step 2**				.075	.017
Gender (1 = *Female,* 2 = *Male*)	−.030	−.022	−.847		
Age	.002	.029	1.111		
Political Ideology	−.051***	−.117***	−4.262		
Shared Humanity	.057***	.119***	4.537		
**Step 3**				.224	.149
Gender (1 = *Female,* 2 = *Male*)	−.030	−.022	−.847		
Age	.002	.029	1.111		
Political Ideology	−.051***	−.117***	−4.262		
Shared Humanity	.057***	.119***	4.537		
Classism	−.294***	−.367***	−14.101		
Awareness of Socio-economic Privilege	.083***	.130***	4.787		

*Note*. ***p < .001, *p < .05

Results indicated that political ideology and gender were significant predictors of support for policies about socio-economic equality in Step 1, *F*(3, 1202) = 24.64, *p <* .001; *R*^2^ = .06. Specifically, people who placed themselves on the left side of the political spectrum (*β* = −.21, *p* < .001) and self-identified as women (*β* = −.07, *p* = .019) reported more support for policies about socio-economic equality. However, age was not a significant predictor of support for policies about socio-economic equality (*β* = .05, *p* = .061).

Identification with all humanity significantly predicted support for policies about socio-economic equality in Step 2, *F*(4, 1201) = 24.39, *p <* .001; *R*^2^ = .08, Δ*R*^2^ = .02; Δ*F* = 22.32, *p <* .001, thus supporting our first hypothesis. In particular, more identification with all humanity (*β* = .12, *p* < .001) predicted more support for policies about socio-economic equality, and political ideology (*β* = −.12, *p* < .001) remained a significant predictor in Step 2 even though its effect dropped.

With respect to the socio-economic factors, classist attitudes (*β* = −.37, *p* < .001) and awareness of socio-economic privilege (*β* = .13, *p* < .001) were significant predictors of support for equal socio-economic policies in Step 3, *F*(6, 1199) = 57.80, *p <* .001; *R*^2^ = .22, Δ*R*^2^ = .15; Δ*F* = 115.35, *p <* .001. In particular, stronger endorsement of classist attitudes predicted less support for equal socio-economic policies; thus, our second hypothesis was supported. Simultaneously, the higher the awareness of socio-economic privilege was perceived, the higher the support for policies about socio-economic equality, therefore supporting our third hypothesis. In addition, after entering the socio-economic beliefs predictors variables in Step 3, the impacts of political ideology (*β* = −.12, *p* < .001) and identification with all humanity (*β* = .12, *p* < .001) remained significant even though their effects dropped.

Study 1 offers initial support for our first hypothesis that identification with all humanity would predict more support for equal socio-economic policies. The findings of Study 1 also supported our two hypotheses regarding socio-economic beliefs. The findings showed socio-economic beliefs, such as people’s higher awareness of class-based privilege, predicted more support for equal socio-economic policies. On the other hand, people’s endorsement of classist attitudes predicted less support for such policies.

We collected data from a convenience sample in Turkey in Study 1. Our participants were mostly young people, who were educated, and left-leaning. Therefore, the participants we presented in Study 1 were not very diverse. Given the potential policy implications of our findings, we addressed this issue in Study 2. We aimed to replicate the findings of Study 1 with a more heterogeneous sample by using a company that specializes in collecting data from relatively more diverse populations regarding ethnicity and geographic locations (i.e., Prolific Academic; see Palan & Schitter, 2018).

## Study 2

In Study 2, we aimed to replicate the findings of Study 1 in a different context (i.e., the U.S.) with a more diverse sample. We chose the U.S. for two reasons. First, even though the U.S. is considered a wealthy country, the wealth gap between North America’s richest and poorest families is increasing fast (Menasce Horowitz, Igielnik, & Kochhar, 2020), and the coronavirus pandemic is making the gap between rich and poor even more apparent. Second, the U.S. has the most expensive health care system in the world. During the early phases of coronavirus, the then president, Donald Trump, said hospitals in the U.S. would be paid for treating uninsured coronavirus patients. However, critics argued that this plan does not address the structural problems of uninsured people more generally (Abelson & Sanger-Katz, 2020). As we aimed to examine people’s support for equal socio-economic policies, including health care, living conditions of the poor, and education (see items above), the U.S. as a context provided us a unique opportunity to test the generalizability of our findings in Study 1.

### Method

#### Participants and Procedure

Participants were recruited on Prolific Academic in May 2020, and they received a payment of 2 US$. The original sample consisted of 225 participants from the U.S. Participants who failed the attention check question^4^ (*N* = 13) were dropped, and the remaining sample consisted of 212 participants. Out of 212 participants, 156 self-identified as White, 19 as Black, 13 as Asian, 10 as Hispanic, 9 as mixed ethnic background, one native American, two chose other, and two did not respond to this question.

One hundred twenty-four respondents self-identified as female, 86 as male, and two as “other.” Respondents’ ages ranged from 18 to 73 years (*M* = 32.34; *SD* = 11.57). Twenty-seven participants completed an MSc/PhD degree, 79 a university degree, 28 an associates degree, 68 high school, one some high school, and five did not complete any high school.

When describing their household income per year, 15 (7%) participants chose *less than $10,000*, 18 (8%) *$10,000–$19,999*, 24 (11%) *$20,000-29,999*, 17 (8%) *$30,000–$39,999*, 18 (8%) *$40,000-49,999*, 12 (6%) *$50.000–$59.999*, 16 (7%) *$60,000–$69,999*, 15 (7%) $*70,000–$79.999*, 5 (2%) *$80,000–$89,999*, 12 (6%) *$90,000–$99,999*, 11 (5%) *$100,000–$109,999*, 8 (4%) *$110,000–$119,999*, 17 (8%) *$120,000–$149,999*, 18 (9%) *$150,000 or more*, and six (3%) preferred not to say.

When describing their socio-economic status, 25 (12%) identified as lower class, 61 (30%) as lower middle class, 85 (40%) as middle class, 37 (17%) as upper-middle class, and 4 (2%) as upper class. When asked to describe their political orientation on a scale from 0 (*liberal*) to 10 (*conservative*), respondents indicated a wide variety of responses across the political spectrum (*M* = 3.28; *SD* = 2.63).^5^ All means and *SD*s are reported in Table 3.

**Table 3:** Means, Standard Deviations, and Correlations among the Study Variables in Study 2

Variables	*M (SD)*	1	2	3	4	5	6	7	8	9	10
1. Gender (1 = *Female*, 2 = *Male*)	–	–	−.157*	.139*	.030	.222**	.070	−.072	.061	−.029	−.117
2. Age	32.34 (11.57)		–	−.015	.157*	−.004	.281***	.013	.117	−.234**	−.083
3. Income	7.22 (4.35)			–	.291***	.671***	.093	.006	.077	−.115	−.217**
4. Education	4.31 (1.23)				–	.240***	.027	.089	−.025	−.029	−.033
5. Subjective SES	2.69 (0.95)					–	.091	.102	.120	−.056	−.189**
6. Political Ideology	3.28 (2.63)						–	−.101	.381***	−.369***	−.473***
7. Shared Humanity	4.54 (1.37)							–	−.137*	.109	.293***
8. Classism	2.00 (1.09)								–	−.261***	−.433***
9. Awareness of Socio-economic Privilege	5.43 (1.07)									–.526***	
10. Support for Policies about Socio-economic Equality	5.67 (1.04)										–

*Note*. ***p < .001, **p < .01, *p < .05

#### Measures

We used the same measures as those used in Study 1 to assess shared humanity (Buchan et al., 2011; Reysen & Katzarska-Miller, 2013; Cronbach’s *α* = 0.85), awareness of socio-economic privilege (Cronbach’s *α* = 0.74), classism (North & Fiske, 2013; Cronbach’s *α* = 0.76) and support for policies about socio-economic equality (Cronbach’s *α* = 0.84). As in Study 1, participants were asked to report the extent they agree with the scale items on 7-point response scales (1 = *strongly disagree/not at all*, 7 = *strongly agree/very much*). We again asked participants’ gender, age, income, education, subjective socio-economic status, and political ideology.

In addition, as in Study 1, to examine the factor structure of our measures, we ran confirmatory factor analyses using IBM Statistics AMOS 23 (Arbuckle, 2014) on data from 212 participants in Study 2. Regarding identification with all humanity, the fit indices for the single factor model was at the acceptable level, except the *RMSEA* value, *χ2*(2, *N* = 212) = 6.478, *p* = .039, *RMSEA* = .103, *GFI* = .984, *AGFI* = .921, *CFI* = .988. Item factor loadings were between .64 and .84. Regarding the awareness of socio-economic privilege measure, however, the single factor model did not yield adequate fit, *χ2*(5, *N* = 212) = 90.410, *p* < .001, *RMSEA* = .285, *GFI* = .876, *AGFI* = .627, *CFI* = .770. Item factor loadings were between .31 and .86. With regard to classism, as in Study 1, the goodness of fit statistics in Study 2 were as follow: χ2(0, *N* = 1212) = 0.00, *CFI* = 1.000. Item factor loadings were between −.51 and .87.^6^ With respect to support for policies about socio-economic equality, the fit indices for the model were at the acceptable level, except the *RMSEA* value χ2(5, *N* = 212) = 16.712, *p* = .005, *RMSEA* = .105, *GFI* = .969, *AGFI* = .906, *CFI* = .977. Item factor loadings were between .55 and .91.

### Results and Discussion

Table 3 presents correlations and other descriptive statistics in Study 2. As only income, subjective SES, and political ideology were significant predictors of support for policies about economic equality, they were included in the regression analysis. As in Study 1, a hierarchical regression analysis^7^ was carried out in SPSS version 26 (IBM Corp, 2017) to examine the degree to which (a) socio-demographic and socio-political characteristics of participants (income, subjective SES and political ideology), (b) shared humanity (identification with all humanity), and (c) beliefs about socio-economic inequality (awareness of socio-economic privilege and classism) predicted support for policies about socio-economic equality. In this analysis, the same steps in Study 1 were followed. The standardized and unstandardized coefficients are presented in Table 4.

**Table 4:** Model of Summary of Regression Analysis in Study 2

Support for Policies about Socio-economic Equality					
	Unstandardized β	Standardized *β*	*t*	*R*^2^	Δ*R*^2^
**Step 1**				.256	.256
Income	−.033	−.137	−1.702		
Subjective SES	−.060	−.055	−.682		
Political Ideology	−.180***	−.455***	−7.575		
**Step 2**				.322	.066
Income	−.026	−.109	−1.409		
Subjective SES	−.112	−.103	−1.322		
Political Ideology	−.169***	−.427***	−7.382		
Shared Humanity	.198***	.261***	4.488		
**Step 3**				.479	.157
Income	−.019	−.078	−1.135		
Subjective SES	−.101	−.093	−1.345		
Political Ideology	−.090***	−.227***	−3.968		
Shared Humanity	.162***	.213***	4.123		
Classism	−.198***	−.208***	−3.735		
Awareness of Socio-economic Privilege	.339***	.351***	6.359		

*Note*. ***p < .001

Results indicated that political ideology was a significant predictor of support for policies about socio-economic equality in Step 1, *F*(3, 208) = 23.82, *p <* .001; *R*^2^ = .26. Specifically, people who placed themselves on the liberal side of the political spectrum reported more support for policies about socio-economic equality (*β* = −.46, *p* < .001). As in Study 1, identification with all humanity significantly predicted support for policies about socio-economic equality in Step 2, *F*(4, 207) = 24.54, *p <* .001; *R*^2^ = .32, Δ*R*^2^ = .07; Δ*F* = 20.14, *p <* .001. In other words, higher identification with all humanity (*β* = .26, *p* < .001) predicted more support for policies about socio-economic equality (confirming our first hypothesis), and political ideology (*β* = −.43, *p* < .001) remained a significant predictor in Step 2.

Similar to Study 1, with respect to the socio-economic beliefs predictors, both classism (*β* = −.21, *p* < .001) and awareness of socio-economic privilege (*β* = .35, *p* < .001) were significant predictors of support for policies about socio-economic equality in Step 3, *F*(6, 205) = 31.40, *p <* .001; *R*^2^ = .48, Δ*R*^2^ = .16; Δ*F* = 30.93, *p <* .001. In particular, the higher the awareness of socio-economic privilege was perceived, and the less classism was endorsed, the higher were support for policies about socio-economic equality (confirming our second and third hypotheses). In addition, after entering the socio-economic beliefs predictors variables in Step 3, political ideology (*β* = −.23, *p* < .001) and identification with all humanity (*β* = .21, *p* < .001) remained significant predictors.

Study 2 replicated the findings in a different context, the U.S. As in Study 1, Study 2 provided support for our hypothesis that stronger identification with all humanity would predict more support for equal socio-economic policies. Similar to Study 1, the findings of Study 2 also supported our hypotheses regarding socio-economic beliefs. We found that socio-economic beliefs such as people’s awareness of class-based privilege predicted more support for socio-economic policies, whereas classist attitudes predicted less support for such policies.

## General Discussion

In the current studies, we aimed to investigate the role of identification with all humanity during the pandemic and beliefs about socio-economic inequality in supporting policies about socio-economic equality. In this contribution, we sought to examine whether people’s identification with humanity and socio-economic factors, such as awareness of class-based privilege and classist attitudes, could become both *facilitators* of and *barriers* to supporting equal socio-economic policies. We proposed that people would support socio-economic policies favoring economic equality in the post-pandemic period to the extent they identify with all humanity in the context of a global crisis that affects all people (*Hypothesis 1*). We also proposed that classist attitudes would negatively predict support for economic equality (*Hypothesis 2*), and awareness of class-based privilege would positively predict support for economic equality (*Hypothesis 3*). We found evidence using correlational designs in two different countries (Turkey and the U.S.) and both studies offered support for our hypotheses. The current investigation extends previous research on shared humanity, classism, and awareness of socio-economic privilege by suggesting that these factors may play important roles as facilitators of and barriers to promoting social and economic justice, particularly in the context of the pandemic.

The results of the present investigation demonstrate the importance of considering shared humanity during the pandemic in understanding support for policies about socio-economic equality. As mentioned earlier, previous research has shown that identification with all humanity is associated with supporting global issues (McFarland et al., 2012) such as supporting (a) refugees and asylum seekers (Bassett & Cleveland, 2019; Nickerson & Louis, 2008), (b) ending global hunger (McFarland et al., 2019), (c) human rights and humanitarian reliefs (McFarland et al., 2012), (d) Fairtrade products over conventional products (Reese & Kohlmann, 2015), and (e) environmental sustainability (Reysen & Katzarska-Miller, 2013). Our set of studies complements previous findings in two ways. First, even though identification with all humanity has been studied concerning global issues like world hunger, refugees, and environmental problems, to our ultimate knowledge, none of these studies examined the role of identification with all humanity in supporting equal economic policies. Second, all of the aforementioned studies in the shared humanity literature were conducted before the coronavirus pandemic. Given the coronavirus pandemic exposed the longstanding inequalities in societies, mainly affected individuals from a low socio-economic background in different parts of the world (e.g., Jetten et al., 2020; Reeves & Rothwell, 2020), our findings highlight the importance of shared humanity in the post-pandemic period. Identification with all humanity may help people endorse a belief that social and economic inequalities are unjust, and that everyone is fundamentally deserving of similar positive outcomes. The way people identify with humanity may also shape their political responses to global challenges (see, e.g., Reese & Kohlmann, 2015). Therefore, in the ongoing pandemic context, identification with all humanity may provide researchers with a framework to explain individual differences in responding to this global challenge (see Hamer et al., 2021).

Our results also showed how people’s beliefs about socio-economic inequality might be predictors of support for equal economic policies. We argued that people’s classist attitudes would negatively predict their support for economic equality (*Hypothesis 2*), and the findings in the two studies supported this hypothesis. As mentioned before, previous research showed that classism has some consequences for supporting socio-economic policies such as reduced funding as well as restrictive and conservative policies (Appelbaum, 2001; Bullock, 1999; Bullock, 2008; Bullock et al., 2003; Kluegel & Smith, 1986). However, less is known on the role of classism in reactions toward economic policies especially after longstanding emergencies such as the pandemic. Given that the pandemic has led to prejudice toward people’s social classes (e.g., Rosen, 2020) such as blaming low-income groups for increasing the virus’s transmission and seeing them as a burden on their health and economic systems (Diallo, 2020; Malik, 2020; Waugh, 2020) and the pandemic has enhanced classism, classist attitudes in the coronavirus pandemic context should be well understood to create equal socio-economic policies in the post-pandemic period. Our results complement previous findings by showing how classist attitudes may be related to less willingness to support equality in health care, living conditions, and education between the economically advantaged and disadvantaged. Despite being correlational, our studies show how classist attitudes could become *barriers* to supporting equal socio-economic policies in the long run.

On the other hand, our findings also showed that higher awareness of class-based privilege predicts more support for economic equality (*Hypothesis 3*), thus confirming our third hypothesis. Even though privilege awareness among advantaged groups has been studied in different intergroup relations such as racial relations (Powell et al., 2005; Stewart et al., 2012; Uluğ & Tropp, 2021) and sexual orientation relations (Case & Stewart, 2010), we know little about the consequences of the status-based privilege awareness or the ways in which socio-economic privilege awareness plays a role in reactions toward socio-economic policies, which may improve the relations between the economically advantaged and economically disadvantaged. Our results contribute to filling this gap in the literature. In addition, in the context of COVID-19, the privileged are less vulnerable than the poor to suffering brought about by the virus (see Jetten et al., 2020) because they can afford to stay home, have secure employment, and better access to health care. As the privileged are less likely to be aware of such benefits and opportunities in general (Dunlap et al., 2007), it is fair to argue that they are also less likely to be aware of those privileges during the pandemic. Albeit correlational, our studies show how awareness of class-based privileges could become a *facilitator* of supporting equal socio-economic policies in the long run.

Taken in conjunction, these findings speak to the utility of considering shared humanity and beliefs about socio-economic inequality in understanding *facilitators* of and *barriers* to supporting socio-economic policies. Beyond its theoretical contributions, the present investigation has the potential to offer some insights to practitioners to increase socio-economic equality. Our findings suggest that people could support equal economic policies to the extent that they 1) identify with all humanity during the pandemic, 2) are aware of their socio-economic status-based privilege, 3) do not hold classist attitudes. If this is the case, community leaders who design and implement interventions and media messages toward reducing socio-economic problems (e.g., economic inequality, unemployment, poverty, etc.) during and after the pandemic should be done in a way that considers such facilitators of and barriers to socio-economic justice (see also Kanık, Solak, & Uluğ, 2020).

### Limitations and Future Directions

Our study has a few limitations. First, we focused on supporting equal socio-economic policies by framing our items as “*over the next few years, I support …*” However, we measured participants’ support for such policies during the ongoing pandemic. Even though our findings may shed some light on people’s beliefs related to class and privilege and attitudes toward such policies in general, we should note that participants’ attitudes may change when it comes to supporting actual policies in the long run, depending heavily on how the course of the pandemic will play out. It is also important to underline that as the pandemic continues, we could not directly assess the pandemic’s influence on participants in the post-pandemic period. The longitudinal studies are needed to examine how the study variables impact support for socio-economic policies before and after the pandemic. Second, we should note that we focused only on social policies that aim to improve disadvantaged groups’ position in society. Future studies may also focus on more restrictive policies, donations, helping behaviors in particular (see, e.g., Tekin et al., 2021). Third, our samples in both studies were mostly young people, who are educated (especially in Study 1), left/liberal-leaning, and mostly lower middle class or middle class (especially in Study 2). Future studies should make a more concerted effort to get a more heterogeneous sample from a) people from high middle class or high class and b) right/conservative-leaning people. Fourth, although we obtained empirical support for our hypotheses across studies, the model fit indices of some of the scales we used were not good enough. Future studies should test our hypotheses using the measures that yield better psychometric properties.

Across two studies, we controlled for six socio-demographic and socio-political variables: gender, age, income, education, subjective SES, and political ideology. After entering all of our study variables, only political ideology remained a significant predictor of support for equal economic policies in both studies. In particular, people who placed themselves on the left side (Study 1) and the liberal side (Study 2) of the political spectrum reported more support for policies about socio-economic equality than those who placed themselves on the right side and the conservative side, respectively. Even though we did not focus on political ideology as a study variable, yet controlled for it, future studies need to take people’s political orientations into account when studying support for economic policies. In particular, given that left/liberal-leaning people are more likely to support equal socio-economic policies as shown in our study (see also Bobbio, 1996), future studies should also focus on the moderating role of political ideology in understanding the association between identification with all humanity and support for economic policies and the moderating role of political ideology in examining the association between classism and support for economic policies.

Finally, the studies have a cross-sectional survey design. The findings limit our confidence in the causal mechanisms identified here between identification with all humanity, socio-economic factors (i.e., awareness of class-based privilege and classist attitudes), and support for equal socio-economic policies. However, it is crucial to examine our hypotheses with more rigorous causal designs, such as experiments. Future studies should be designed to establish the causal order between shared humanity, awareness of class-based privilege, and classist attitudes and support for such policies to understand which one causes the other. By examining the role of shared humanism and beliefs about socio-economic inequality, the present investigation may pave the way for understanding and perhaps overcoming the barriers that may stand in the way of equal economic policies in the post-pandemic period.

## Footnotes

1 One instructional attention check question was used (see Oppenheimer, Meyvis, & Davidenko, 2009).

2 In Study 1, we ran Exploratory Factor Analysis (EFA) for the classism scale to see the factor loadings and explained variance of the items. Principal Component Analysis results showed that there is only one component (eigenvalue 1.833). This component explains 61.1% of the variance and factor loadings of the items are .86, .87, .58, respectively.

3 We tested the regression assumptions in Study 1 before we analyzed the data. The results showed that there is no multicollinearity in our data (all VIF scores < 10 and tolerance scores > .02), the values of the residuals are independent (the Durbin-Watson score of 1.09 is in the range of DW > 1 and < 3), and the values of the residuals in the data are normally distributed. Last, we should note that our plot of standardized residuals vs. standardized predicted values suggests that the assumption of homoscedasticity may have been violated.

4 As in Study 1, we used instructional attention check questions (two questions; see Oppenheimer et al., 2009).

5 As the scaling 1–5 for the socio-economic status (see, e.g., Tropp & Uluğ, 2019) and 0–10 for the political ideology (e.g., Thorisdottir, Jost, Liviatan, & Shrout, 2007) are much more common in the U.S., we decided to use these scaling types in Study 2.

6 In Study 2, we ran Exploratory Factor Analysis (EFA) for the classism scale to see the factor loadings and explained variance of the items. Principal Component Analysis results showed that there is only one component (eigenvalue 2.048). This component explains 68.3% of the variance and factor loadings of the items are .87, .88, .71, respectively.

7 We again tested the regression assumptions in Study 2 before we analyzed the data. The results showed that there is no multicollinearity in our data (all VIF scores < 10 and tolerance scores > .02), the values of the residuals are independent (the Durbin-Watson score of 1.91 is in the range of DW > 1 and < 3), and the values of the residuals in the data are normally distributed. Last, we should note that our plot of standardized residuals vs. standardized predicted values suggests that the assumption of homoscedasticity may have been violated.
